# 4-1BBL Enhances CD8^+^ T Cell Responses Induced by Vectored Vaccines in Mice but Fails to Improve Immunogenicity in Rhesus Macaques

**DOI:** 10.1371/journal.pone.0105520

**Published:** 2014-08-20

**Authors:** Alexandra J. Spencer, Julie Furze, Jared D. Honeycutt, Alice Calvert, Saroj Saurya, Stefano Colloca, David H. Wyllie, Sarah C. Gilbert, Migena Bregu, Matthew G. Cottingham, Adrian V. S. Hill

**Affiliations:** 1 The Jenner Institute, University of Oxford, Oxford, United Kingdom; 2 Okairòs, Rome, Italy; Federal University of São Paulo, Brazil

## Abstract

T cells play a central role in the immune response to many of the world’s major infectious diseases. In this study we investigated the tumour necrosis factor receptor superfamily costimulatory molecule, 4-1BBL (CD137L, TNFSF9), for its ability to increase T cell immunogenicity induced by a variety of recombinant vectored vaccines. To efficiently test this hypothesis, we assessed a number of promoters and developed a stable bi-cistronic vector expressing both the antigen and adjuvant. Co-expression of 4-1BBL, together with our model antigen TIP, was shown to increase the frequency of murine antigen-specific IFN-γ secreting CD8^+^ T cells in three vector platforms examined. Enhancement of the response was not limited by co-expression with the antigen, as an increase in CD8^+^ immunogenicity was also observed by co-administration of two vectors each expressing only the antigen or adjuvant. However, when this regimen was tested in non-human primates using a clinical malaria vaccine candidate, no adjuvant effect of 4-1BBL was observed limiting its potential use as a single adjuvant for translation into a clinical vaccine.

## Introduction

Since the development of the first vaccine by Edward Jenner, vaccination has made a substantial impact on the burden of disease worldwide. While traditional vaccination with heat-killed or attenuated vaccines has proved highly effective against pathogens controlled by neutralising antibodies, very few vaccines been licensed against pathogens in which cell mediated immunity plays a primary role in protection. In recent years, viral vectored vaccines have demonstrated a remarkable capacity to induce and boost T cell immunity and leading to their development for a number of human and veterinary vaccines [1].

Malaria kills around 0.65 million individuals per annum, mostly in sub-Saharan Africa and primarily in children under the age of 5 [2]. Cell mediated protection against the liver-stage of disease, the first stage of infection in murine or human host, was originally demonstrated in mice following administration of irradiated sporozoites and this was shown to be mediated by CD8^+^ T cells [3]–[5]. This led to the development of vectored vaccines to specifically induce CD8^+^ T cells to kill infected hepatocytes [6]. The highest level of IFN-γ producing CD8^+^ cells induced by vaccination with the lead subunit antigen ME-TRAP, *Plasmodium falciparum* TRAP (thrombospodin related adhesion protein) fused to ME, a string of 20 malarial T- and B-cell epitope [7], has been observed with prime-boost regimen employing a recombinant chimpanzee adenovirus 63 (ChAd63) vector followed by recombinant modified vaccinia virus Ankara (MVA) [8]–[10]. However to achieve sufficient protection against malaria, even higher levels of antigen specific T cells are likely required [11].

Complete T cell activation is only achieved when binding of the antigen-MHC complex to the T cell receptor (TCR) occurs in conjunction with a second costimulatory signal [12], [13]. To ensure tolerance to peripheral self-antigens, yet maximising T cell activation after pathogenic stimulation, both costimulatory molecules and their corresponding ligands have tightly regulated expression patterns, and therefore play a critical role in shaping the size and quality of the T cell response. In the tumour necrosis factor receptor (TNFR) superfamily, 4-1BB interactions with its ligand 4-1BBL (a.k.a. CD137L and TNFSF9) are capable of inducing bidirectional positive signalling resulting in increased cytokine production by both CD8^+^ T cells and dendritic cells (DCs) [14], [15] and increase in T cell proliferation [16]–[18]. In the absence of 4-1BB/4-1BBL signalling however, antigen specific CD8^+^ T cell responses are reduced [19]–[21], particularly recall responses due to decreased cell survival [22]. Due to the selective expression of 4-1BB only after TCR activation [18], [22], [23], increasing 4-1BB:4-1BBL signalling has been investigated as a mechanism to increase antigen specific CD8^+^ T cell levels, while avoiding non-specific activation of naive cells [24].

A number of different studies have documented an increase in immunogenicity by administration of a monoclonal 4-1BB agonist [25]–[29], while *in vivo* administration of DCs expressing antigen and 4-1BBL [30]–[32] or vaccination with vectored vaccines expressing 4-1BBL [33]–[36] has been studied as an alternative method to increase CD8^+^ T cells. While daily administration of a 4-1BB agonist may be feasible in a therapeutic vaccination setting, the major target population of a malaria vaccine comprises children in rural communities, and therefore only simple vaccine preparations that align with the EPI regimen will be deployable.

In this study we designed two approaches to investigate whether 4-1BBL would enhance T cell immunogenicity when encoded by vectored vaccines. We investigated whether expression of 4-1BBL from a DNA plasmid vaccine and two viral vectored vaccines, non-replicating human adenovirus type 5 (HAdV5) and the attenuated poxvirus MVA, could enhance vaccine induced immune responses to a transgenic antigen. To maximise the potential augmentation of the response for the murine studies, we initially chose a vaccine system in which our model antigen would be co-expressed by the same vector. The second approach was to investigate whether the co-administration of a 4-1BBL encoding vector with an antigen encoding vector would enhance T cell immunogenicity. This regimen would make possible a more flexible clinical deployment where the adjuvant-expressing virus could be mixed with a number of existing clinical vaccines for various disease indications. In mice, 4-1BBL was also shown to increase the CD8^+^ T cell response when mixed in excess with a separate antigen-expressing vaccine. This encouraged further testing of the coadministration approach in non-human primates using a clinical malaria vaccine ChAd63.ME-TRAP. Since no adjuvant effect of 4-1BBL was observed in rhesus macaques, this has halted the translation of this single adjuvant approach for a clinical vaccine and highlights the importance of testing novel adjuvant approaches in higher order species.

## Results

### Genetic instability of dual CMV major immediate-early promoters in adenoviral vectors

**Figure 1:**
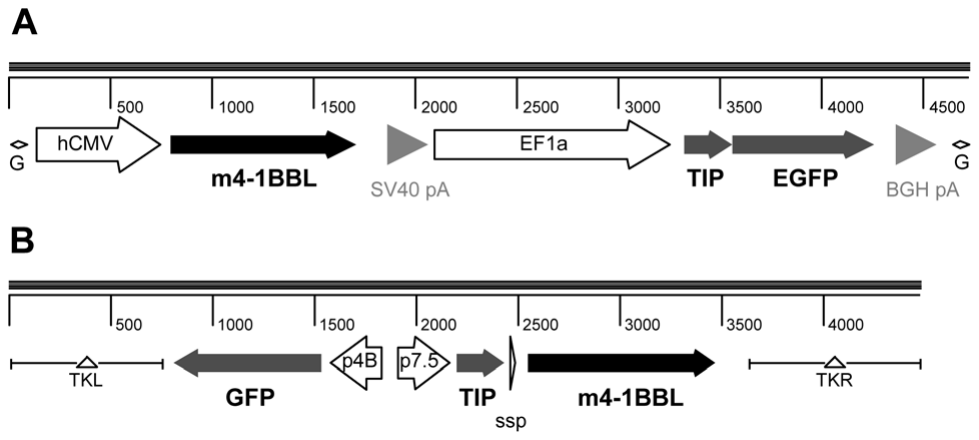
Schematic of cassettes inserted into recombinant adenovirus and MVA vectors. **A)** Bi-cistronic transgene expression cassette for insertion into E1 locus of HAdV5 vector. A human CMV immediate early promoter (CMV) drives expression of the mouse 4-1BBL open reading frame, and the human EF1α promoter (EF1a) drives expression of the TIP-EGFP fusion protein, used as a model antigen. Two different polyadenylation signals (pA) are used. This assembly is flanked by *AttL* sites for Gateway cloning (G) into the HAdV-5 vector genomic clone. Ruler is in nucleotides. **B)** Tri-cistronic transgene expression cassette for insertion into TK locus of MVA vector. The early-late vaccinia virus p7.5 promoter drives expression of the TIP model antigen (not fused to GFP) and the early-late short synthetic promoter (SSP) drives expression of mouse 4-1BBL, while the late fowlpox virus p4B promoter drives expression of the GFP marker gene. This assembly is flanked by sequences from the viral thymidine kinase locus (TKL and TKR) to allow recombination into the viral genome in infected cells. Ruler is in nucleotides.

Based on the hypothesis that co-expression of 4-1BBL and a transgenic antigen from a single infected cell (and therefore from the same vector) would be the most sensitive system for detection of an immunopotentiating effect of 4-1BBL, we adopted a bi-cistronic approach, employing two tandem transgenic expression cassettes, one for each open reading frame. We used a model antigen TIP comprising of a epitope string of CD4^+^ and CD8^+^ T cell epitopes [37] in DNA and modified vaccinia virus Ankara (MVA) vectors; and TIP-EGFP, the same antigen fused to enhanced green fluorescent protein (EGFP), in E1/E3-deleted human adenovirus 5 (HAdV5) vectors (Figure 1).

For DNA plasmid vaccines, tandem human cytomegalovirus major immediate early promoters (phCMV) were utilised, each with its own polyadenylation signal (polyA) [37]. Yet when such dual-phCMV cassettes (which are stably propagable in *E. coli*) were inserted into adenoviral vectors, genetic instability resulting from homologous recombination between the identical promoters was observed (Figure S1), as has also been subsequently reported by others [38]. Use of the major immediate early promoter of simian cytomegalovirus [39], which shares ∼61% nucleotide identity with phCMV, did not allow this problem to be circumvented: a tandem phCMV-psCMV arrangement at the adenoviral E1 locus also readily recombined (Figure S1). For the purposes of proof-of-concept mouse studies, we therefore utilized the human elongation factor 1α promoter (phEF1) to drive expression of the TIP-EGFP antigen in tandem with phCMV to direct murine 4-1BBL (m4-1BBL) expression in the E1 locus of HAdV5, an arrangement that overcame issues of genetic instability. No similar issues were encountered in recombinant MVA constructed with TIP and m4-1BBL under control of separate non-identical poxviral early/late promoters inserted at the viral thymidine kinase locus.

### Co-expression of 4-1BBL and a transgenic antigen in DNA plasmid, MVA and HAdV5 vectored vaccines enhances mouse CD8^+^ T cell responses

**Figure 2:**
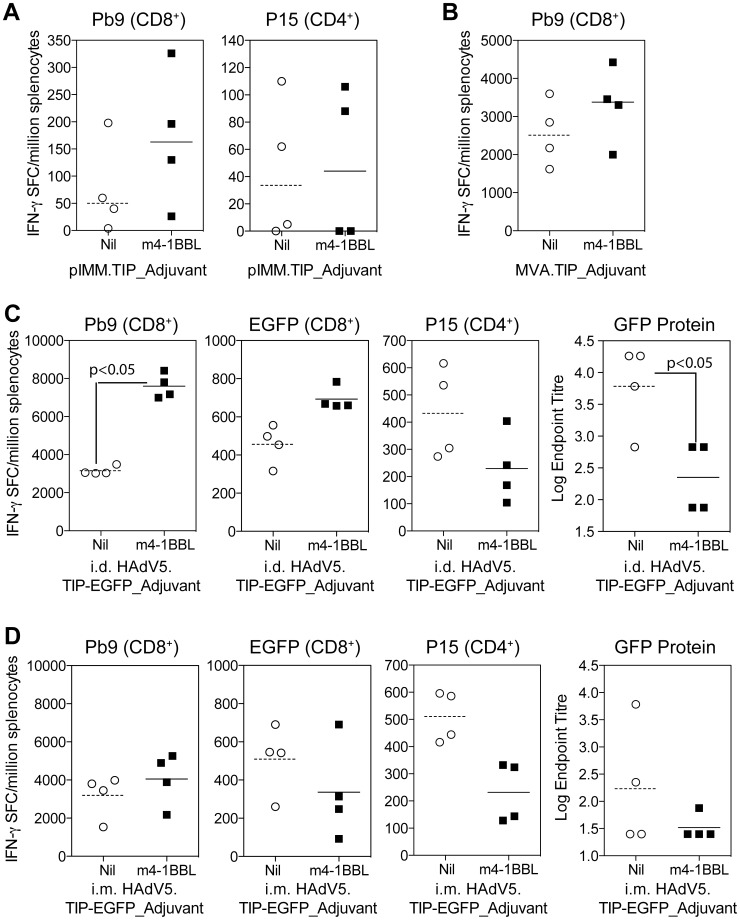
Screening of vectors in Balb/c mice. **A**) Balb/c mice were immunised i.m. with 50 µg of DNA plasmid pIMM.TIP or pIMM.TIP_m4-1BBL, spleens harvested at day 7 and the response to Pb9 and P15 measured by ELISpot. **B**) Balb/c mice were immunised i.d. with 10^6^ PFU of MVA.TIP or MVA.TIP_m4-1BBL, spleens were harvested 1 week later and the response to Pb9 measured by ELISpot. Responses to P15 were not above background levels. **C & D**) Balb/c mice were immunised either i.d. (**C**) or i.m. (**D**) with 10^8^ iu of HAdV5.TIP-EGFP or HAdV5.TIP-EGFP. Spleens and serum were harvested 2 weeks later and the response to Pb9, EGFP and P15 measured by ELISpot and antibodies to GFP protein measured by ELISA. Data was analyzed with a 2-way analysis of variance with a post-hoc Bonferroni test; p values denote the level of statistical significance. For all graphs, the bar represents the median per group with each individual animal displayed as a single point.

To determine whether co-expression of murine 4-1BBL in these three bi-cistronic vectored vaccines could enhance immunogenicity, BALB/c mice were immunised with DNA plasmid pIMM.TIP_m4-1BBL, MVA.TIP_m4-1BBL or HAdV5.TIP-EGFP_m4-1BBL or cognate control vectors lacking the m4-1BBL open reading frame (ORF). After a single immunisation with DNA (i.m.) (Figure 2A) or MVA (i.d.) (Figure 2B), there was a modest (but not significant) increase in the overall response to the CD8^+^ T cell epitope Pb9 in animals vaccinated with TIP_m4-1BBL when compared to the control group immunised with TIP (Figure 2A). With HAdV5 intradermal (i.d.) immunization, we observed an increase in the response to CD8^+^ T cell epitopes Pb9 (p<0.05) and EGFP_200–208_ (not significant) (Figure 2C) in TIP-EGFP_m4-1BBL vaccinated animals compared to TIP-EGFP controls. Interestingly, the increase was limited to CD8^+^ T cell responses as a decrease in both the CD4^+^ T cell epitope P15 and antibodies to GFP were observed. When the same dose of HAdV5 vaccines were administered intramuscularly (i.m.), a small increase in Pb9 responses accompanied by a decrease in CD4^+^ T cells (p<0.05) and antibodies titres was again observed, however no increase in EGFP_200–208_ responses were seen via this route of immunisation (Figure 2D).

Previous reports had suggested that the adjuvant effect of m4-1BBL in poxviral vectors was greater after a boosting vaccination [35], and we therefore set out to determine whether co-expression of m4-1BBL in the prime, boost or in both vaccinations could further increase the level of T cell immunogenicity. Two heterologous prime-boost regimens were investigated: DNA followed two weeks later by MVA or HAdV5 followed eight weeks later by MVA. For both prime-boost vaccine regimens, the greatest enhancement in the Pb9 specific response was observed in animals where m4-1BBL had been expressed in both the priming and boosting vaccine when compared to a regimen with only TIP at either vaccination (Figure S2A). This same trend was also observed when mice received a priming immunisation with HAdV5.TIP-EGFP_m4-1BBL followed by MVA.TIP_m4-1BBL were compared to the non-adjuvanted control group (Figure S2B), resulting in a 3.3 fold increase in the mean group response.

### m4-1BBL does not alter the quality of the response

**Figure 3:**
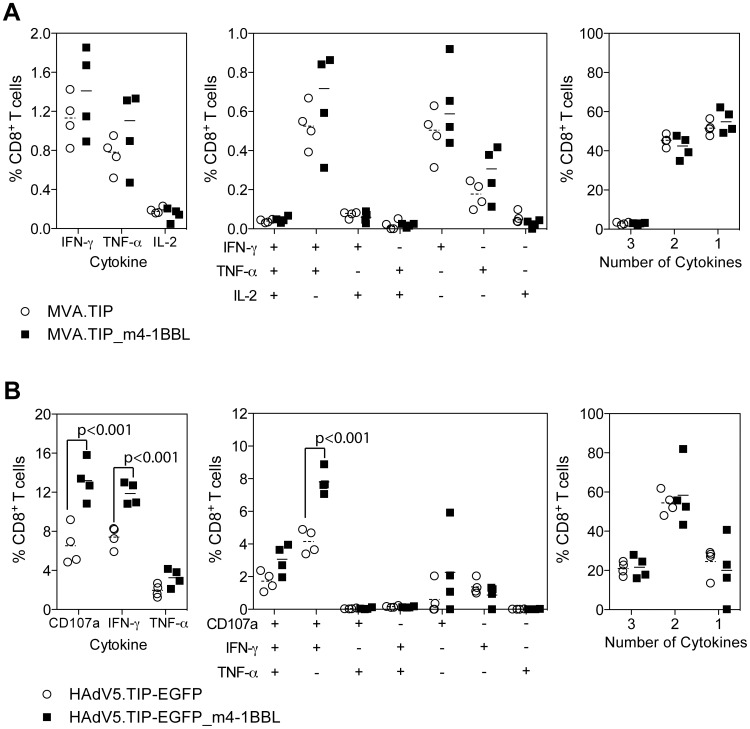
Quality of the CD8^+^ T cell response. Balb/c mice immunised id with 10^6^ MVA.TIP Adjuvant (**A**) or 10^8^iu HAdV5.TIP-EGFP Adjuvant (**B**) and the response measured by intracellular cytokine staining after 6 hours or restimulation with Pb9 peptide. Graphs represent the total frequency of CD8^+^ T cells producing each cytokine (left), each combination of cytokines (central) or the percentage of cytokine producing cells secreting 3, 2 or 1 cytokine (right). For all graphs bars represent the median, with each individual animal displayed as a single point. Each data set was analyzed with a 2-way analysis of variance and a post-hoc Bonferroni test; p values denote the level of statistical significance.

With its reported role in cytokine production, together with studies suggesting the quality of a T cell response might be a useful predictor of vaccine efficacy [40], we wished to determine the effect of coexpression of m4-1BBL on the cytokine phenotype of the T cells response. Spleens taken from Balb/c mice 7 days after vaccination with 10^6^ PFU of MVA.TIP or MVA.TIP_m4-1BBL were analysed for simultaneous production of IFN-γ, TNF-a and IL-2 by intracellular cytokine staining. Analysis of each individual cytokine alone, demonstrated a small increase (but not significant) in the frequency of IFN-γ^+^ or TNF-α^+^ CD8^+^ cells restimulated with Pb9 peptide for 6 hours, but not IL-2^+^ CD8^+^ T cells which was low in both groups (Figure 3A left panel). Subdividing the response into each possible combination of cytokines, demonstrated a domination of the response by IFN-γ and TNF-α double producers, IFN-γ only or TNF-α only producing cells (Figure 3A central panel), with a small increase in each population when animals were vaccinated with MVA.TIP_m4-1BBL. Despite an increase the frequency of cytokine producing cells, the proportion of the cytokine^+^ population able to produce 3, 2, or 1 cytokine was unchanged (Figure 3A right panel).

Frozen spleen samples from mice vaccinated i.d. with 10^8^ iu of HAdV5.TIP-EGFP (Figure 2) or HAdV5.TIP-EGFP_m4-1BBL were analysed for upregulation of degranulation marker CD107a and intracellular production of IFN-γ and TNF-α after 6 hour restimulation with Pb9 peptide. A statistically significant increase in the frequency of CD107a^+^ or IFN-γ^+^ CD8^+^ cells was observed in animals vaccinated with HAdV5.TIP-EGFP_m4-1BBL compared to the control group (Figure 3B left panel). A statistically significant increase in CD107a^+^, IFN-γ^+^ CD8^+^ cells was also observed on further subdivision of the cytokine response (Figure 3B central panel). The overall proportion of the cytokine response producing 3, 2 or 1 cytokine was unchanged between HAdV5.TIP-EGFP and HAdV5.TIP-EGFP_m4-1BBL vaccinated animals.

In summary, coexpression of m4-1BBL induced an increase in CD8^+^ T cell responses by all vectored vaccines platforms tested, most importantly without altering the cytokine profile.

### m4-1BBL co-expression improves the efficacy of HAdV5.TIP-EGFP against mouse liver stage malaria

CD8^+^ T cells play an essential role in control of liver-stage malaria and the dominant epitope from *P.berghei* CS antigen (Pb9), expressed in our antigen TIP, alone can mediate protection in Balb/c mice [41], [42]. To determine whether an increase in Pb9-specific cells by coexpression of 4-1BBL would reflect in an increase in protection, mice were challenged with a lethal dose of *P.berghei* and monitored for development of blood-stage malaria. As there are no other *P.berghei* antigenic determinants present in our model antigen TIP, we were able to use linear regression of blood-stage malaria growth to calculate the time at which mice reached a threshold level of malaria, set at 0.5%.

**Figure 4:**
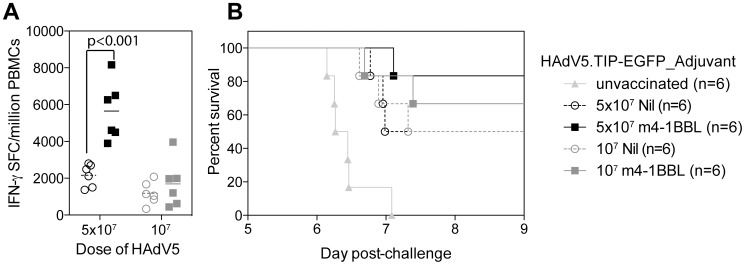
Challenge with *P.berghei* sporozoites. Balb/c mice (6 mice per group) were immunised i.m. with two different doses of HAdV5.TIP-EGFP or HAdV5.TIP-EGFP_m4-1BBL and challenged with 1000 *P.berghei* sporozoites 2 weeks later. The response to Pb9 in the blood was measured 2 days prior to challenge by IFN-γ ELISpot. **A)** The bar represent the median with each individual animal displayed as a single point. Data was analysed with a 2-way analysis of variance a post-hoc Bonferroni test; the p value denotes the level of statistical significance. **B)** Parasitaemia was measured daily and the time to 0.5% parasitaemia calculated by linear regression. Survival curves were compared with a log-rank (Mantel-Cox) test but no significant difference between groups was observed.

Balb/c mice were immunised with 5×10^7^ or 10^7^ iu of HAdV5.TIP-EGFP or HAdV5.TIP-EGFP_m4-1BBL and challenged with *P.berghei* sporozoites two weeks after vaccination. IFN-γ ELISpot assays performed on PBMCs prior to challenge once again demonstrated a statistically significant (p<0.001) increase in the immune response by coexpression of m4-1BBL (Figure 4A) for the high dose only, at the lower dose there was only a non-significant trend. At both vaccination doses, more mice vaccinated with HAdV5.TIP-EGFP_m4-1BBL were sterilely protected from malaria challenge than TIP-EGFP vaccinated animals (5×10^7^ 83% vs 50%, 10^7^ 66% vs 50%, not significant by fishers exact test), while those animals which did succumb to malaria had a small (but not significant) delay in the time to 0.5% parasitaemia (Figure 4B). In conclusion, vaccination with TIP-EGFP coexpressing 4-1BBL induced a modest (albeit not significant) increase in survival when animals were challenged with *P.berghei* sporozoites.

### Co-administration of two adenoviral vectors expressing 4-1BBL or a transgenic antigen improves cellular immunogenicity

Having observed enhancement of cellular immune responses induced by three different vectored vaccines by co-expression of m4-1BBL in mice, we next aimed to demonstrate a similar effect of human 4-1BBL (h4-1BBL) in rhesus macaques using a vector suitable for deployment in a possible subsequent comparative human clinical trial. For this purpose, ChAd63.ME-TRAP, a chimpanzee adenovirus vectored liver stage malaria vaccine which has recently been shown to be highly immunogenic in humans [8], was selected as the comparator. The rationale for selection of an adenoviral vector was twofold: (i) we observed the most striking enhancement of immunogenicity with HAdV5 in mice; and (ii) adenoviral vectors are the most immunogenic, as a single-shot regimen, of the three vector platforms tested here.

Like most adenovirus vectored vaccines, ChAd63.ME-TRAP employs phCMV to drive recombinant antigen expression. We considered that it would not be justifiable to alter the promoter (or any other part) of the ME-TRAP expression cassette, since this could itself affect immunogenicity of the comparator vaccine. Indeed, even slightly different variants of phCMV exhibit differential immunogenicity [43], [44]. For the same reason, we did not investigate, for example, internal ribosome entry sites or picornaviral 2A sequences. Furthermore, from a regulatory point of view, a human promoter, such as phEF1 (as used in the bi-cistronic HAdV5 described above) would be undesirable in a malaria vaccine ultimately destined for use in human infants, due to theoretical safety concerns over possible genomic integration of the vector. We therefore investigated whether immunopotentiation by 4-1BBL could be achieved following vaccination with a mixture of two separate adenoviral vectors, one expressing 4-1BBL and one expressing a recombinant antigen, as an alternative route towards clinical testing.

**Figure 5:**
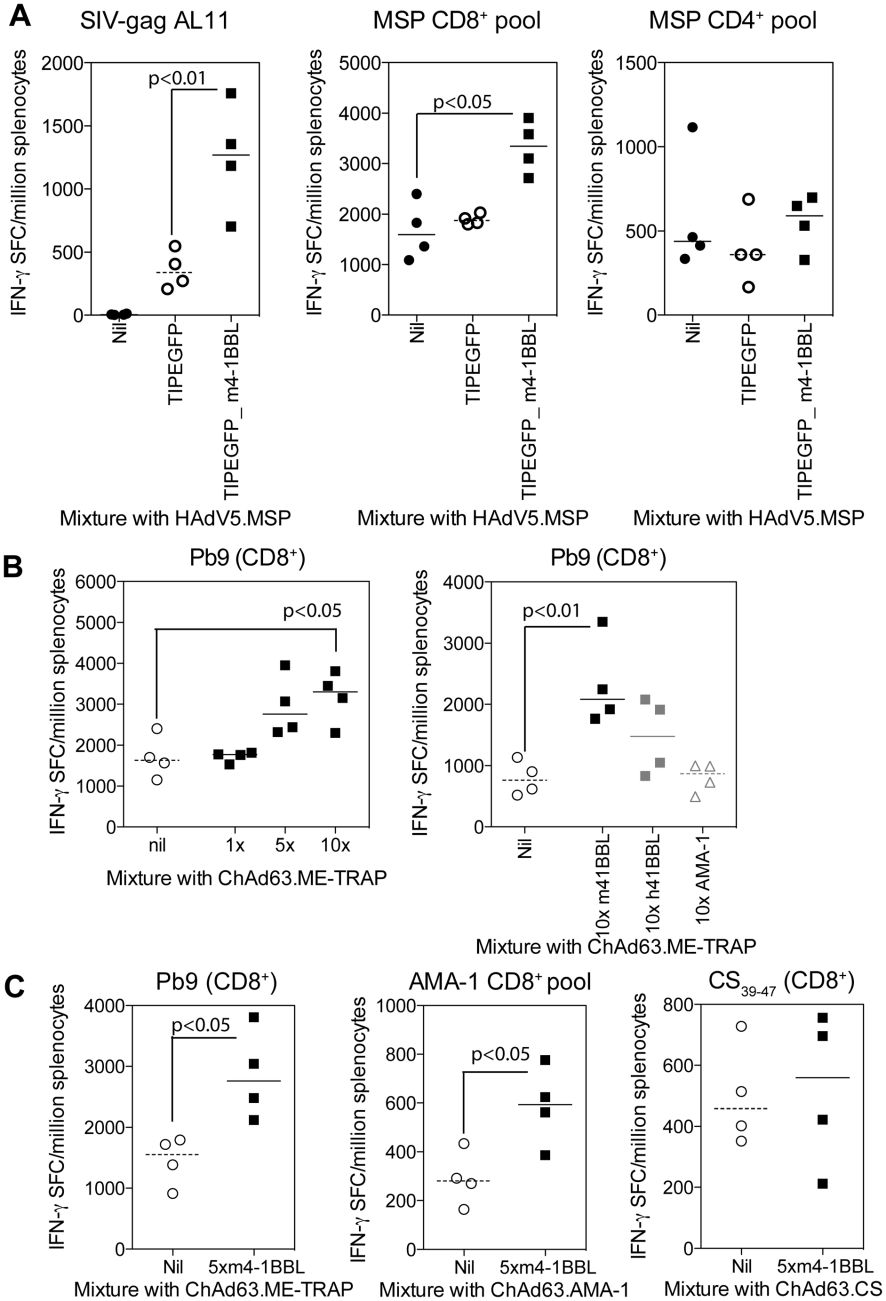
Coadministration of vectors expressing 4-1BBL. **A**) C57BL/6J mice were vaccinated i.m. with 2.8×10^6^ iu of HAdV5.MSP either alone or mixed with 10^7^iu of HAdV5.TIP-EGFP or HAdV5.TIP-EGFP 4-1BBL. Spleens were harvested 2 weeks later and the response to TIP antigen SIV, a pool of CD8^+^ T cell epitopes from MSP or a pool of CD4^+^ T cell epitopes from MSP measured by IFN-γ ELISpot. **B**) In separate experiments, Balb/c mice were vaccinated i.m. with 10^6^ iu of ChAd63.ME-TRAP mixed together with different ratios of ChAd63-m4-1BBL (left) or with 10^7^iu of ChAd63.m4-1BBL, ChAd63.h4-1BBL or ChAd63.AMA-1 (right). Spleens were harvested 2 weeks later and the response to Pb9 measured by IFN-γ ELISpot. **C**) Balb/c mice were immunised i.m. with 10^6^iu ChAd63.ME-TRAP, ChAd63.AMA-1 or ChAd63.CS with or without 10^7^iu ChAd63.m4-1BBL. Spleens were harvested 2 weeks later and antigen specific IFN-γ responses to the relevant antigens measured by ELISpot. For all experiments bars represent the median per group with each individual animal displayed as a single point. Each experiment was analysed with a one-way analysis of variance a post-hoc Bonferroni test (**A**&**B**) or an unpaired t-test (**C**); the p values denote the level of statistical significance.

To this end, we first used the existing bi-cistronic HAdV5 viruses mixed with an additional HAdV5 vaccine. C57BL/6J mice were immunised with 2.8×10^6^ iu HAdV5 expressing the blood stage malaria antigen merozoite surface protein (MSP-1) either alone, or mixed with 10^7^ iu of HAdV5.TIP-EGFP or 10^7^ iu HAdV5.TIP-EGFP_m4-1BBL, and the responses to both TIP and MSP-1 were measured by IFN-γ ELISpot 14 days later. Consistent with previous experiments, vaccination with HAdV5.TIP-EGFP co-expressing m4-1BBL elicited increased an immune response to the TIP-encoded AL11 epitope derived from SIV-gag (Figure 5A left panel) compared to control. The IFN-γ response following restimulation with a pool of overlapping peptides corresponding to MSP-1 demonstrated that coadministration of HAdV5.TIP-EGFP_m4-1BBL significantly increased the MSP-1 specific CD8^+^ T cell response (Figure 5A central panel). In contrast, a comparator vaccination with a mixture lacking the adjuvant HAdV5.TIP-EGFP and HAdV5.MSP1 did not alter the frequency of MSP-1 specific CD8^+^ T cells. No change in CD4^+^ T cell response (Figure 5A right) or MSP-1 specific antibodies (data not shown) was observed between any groups.

To enable the evaluation of 4-1BBL using a clinically-deployable chimpanzee adenoviral vector in this mixing approach, mono-cistronic ChAd63 vectors expressing either m4-1BBL or h4-1BBL, driven by phCMV, were constructed, and their ability to enhance the immune response to ChAd63.ME-TRAP when co-administered as a mixture of two vectors was assessed. Balb/c mice were immunised with 10^6^ iu of ChAd63.ME-TRAP mixed with 10^6^, 5×10^6^ or 10^7^ iu of ChAd63.m4-1BBL and the response to Pb9 (which is present in the multi-epitope string of this antigen) was measured in the spleens of mice 14 days later by IFN-γ ELISpot. With a 5× or 10× ratio of m4-1BBL to ME-TRAP, an increase in the response to Pb9 was observed, while an equal ratio dose of coadministered vaccines did not change the immunogenicity of ME-TRAP (Figure 5B left panel).

In a subsequent experiment, ChAd63 expressing either m4-1BBL, h4-1BBL or an irrelevant molecule, was coadministered to Balb/c mice mixed at a 5× or 10× ratio with 10^6^ iu of ChAd63.ME-TRAP. Consistent with the previous experiment, an increase in Pb9 specific responses was detected with 10× administration of m4-1BBL (Figure 5B right), but only a modest increase (not significant compared to either the control of m4-BBL group) in the response with 10× h4-1BBL was observed. Importantly, no change in the immune response was observed by 10× addition of ChAd63.AMA-1 (an irrelevant malarial protein, apical merozoite antigen 1, serving as a control), demonstrating that mixing *per se* with an additional vector did not affect immunogenicity. In a separate experiment we investigated whether immunisation with m4-1BBL or h4-1BBL resulted in generation of potentially auto-reactive T cells by measuring the IFN-γ ELISpot response to a pool of peptide spanning the length of either mouse or human 4-1BBL. Importantly, mice vaccinated with a mixture containing ChAd63.m4-1BBL demonstrated no significant responses to mouse or human 4-1BBL peptides (Figure S3A). Responses to human 4-1BBL peptides were observed in all animals vaccinated ChAd63.h4-1BBL (Figure S3B), which may, at least in part, explain the reduced efficiency of human 4-1BBL compared to mouse 4-1BBL to enhance the response in mice.

We further investigated the adjuvant effect of murine 4-1BBL with additional *P.falciparum* clinical candidate antigens, namely AMA-1 and circumsporozoite protein (CS). Balb/c mice were immunised with 10^6^ iu ChAd63.ME-TRAP, ChAd63.AMA-1 or ChAd63.CS either alone or mixed together with 5×10^6^ ChAd63.m4-1BBL, and the antigen specific responses measured 14 days later by IFN-γ ELISpot. Consistent with the previous experiment, coadministration of murine 4-1BBL augmented the Pb9-specific response induced by ME-TRAP vaccination (Figure 5C left). In this same experiment, the immune response to AMA-1 was increased by coadministration of murine 4-1BBL (Figure 5C central); however, the response to CS was unchanged by addition of murine 4-1BBL (Figure 5C right). In summary, coadministration of ChAd63.m4-1BBL was shown to increase the immune response to two different malaria candidate antigens delivered by ChAd63, while ChAd63.h4-1BBL only induced only a modest increase in mice.

### Coadministration of ChAd63.h4-1BBL is not sufficient to increase the IFN-γ response in non-human primates

Following on from a consistent increase observed in mice by either coexpression or coadministration of m4-1BBL multiple viral vectors and multiple antigens, it was important to determine whether this adjuvant effect could translate to h4-1BBL in non-human primates.

**Figure 6:**
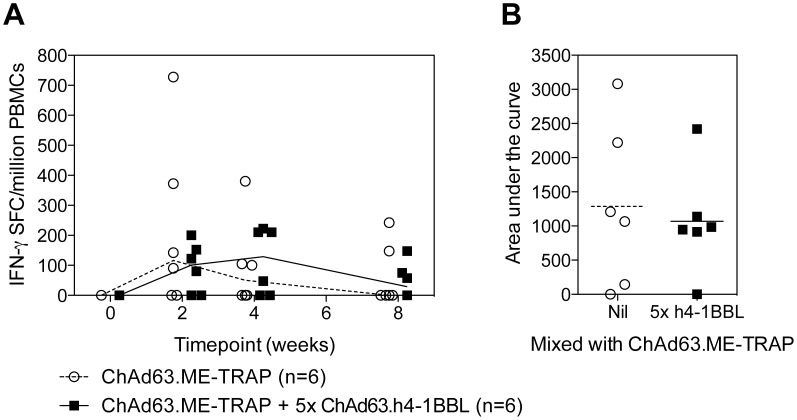
IFN-γ ELISpot responses in rhesus macaques. Male rhesus macaques were immunised i.m. with 10^8^iu ChAd63.ME-TRAP alone or mixed together with 5×10^8^iu ChAd63.h4-1BBL. Blood samples were taken on the day of vaccination and at 2, 4 and 8 weeks post-vaccination and the response to TRAP measured by IFN-γ ELISpot. Graphs represent the total TRAP response at each timepoint (**A**) or the area under the curve of the TRAP response (**B**). For each graph the line or bar represents the median response with each individual animal displayed as a single point.

Male rhesus macaques aged between 4 ½ to 8 ½ years were vaccinated i.m. with 2.75×10^8^ iu of ChAd63.ME-TRAP alone or mixed together with 1.38×10^9^ iu ChAd63.h4-1BBL (5× ratio). Blood samples were taken at 0, 2, 4, 8 weeks and the responses to peptide pools containing ME antigens, or pools of overlapping peptides spanning the length of TRAP and h4-1BBL measured by IFN-γ ELISpot. Consistent with previous studies, the peak of the immune response after ChAd63 was observed 2 weeks after vaccination. At this time point, no difference in immune responses between animals vaccinated with ChAd63.ME-TRAP and the same vaccine with an additional 5× ratio of admixed ChAd63.h4-1BBL was observed (Figure 6A). An area under the curve analysis of each individual animal’s response demonstrated a similar overall level of immunogenicity between the two groups of macaques (Figure 6B). No responses above background were observed against h4-1BBL peptides in any animal or at any time point (data not shown). In summary, despite a promising adjuvant effect of m4-1BBL observed in mice, no adjuvant effect of h4-1BBL was observed in non-human primates.

## Discussion

T cells play a critical role in protection against the liver-stage of malaria and vectored vaccines have shown a remarkable capacity to induce and boost antigen specific cells to a number of malaria antigens. Recent clinical trials have demonstrated the protective capacity of these viral vectored [6], [45], [46] in a proportion of vaccinees, but even higher T cell responses are most likely required to achieve greater levels of efficacy. In an attempt to increase the T cell response induced by vectored vaccination, we assessed the adjuvant capacity of 4-1BBL, a known stimulator of CD8^+^ T cells.

To circumvent adenoviral vector instability due to expression of the antigen and adjuvant driven by dual CMV major immediate-early promoters (whether from human or simian CMV), we used the human EF-1α promoter in a bicistronic HAdV5 for mouse studies, even though we considered that this promoter would not be suitable for clinical translation of the approach. We therefore confirmed and extended the findings with bi-cistronic HAdV5 using mixtures of two HAdV5 vectors or two ChAd63 vectors, before ultimately testing the latter approach in non-human primates.

After encouraging results in the murine system, we were disappointed to observe no change in immunogenicity when rhesus macaques received a vaccination regimen containing ChAd63.ME-TRAP together with 5× ChAd63.h4-1BBL. One may hypothesis that the lack of adjuvant effect was due to different species specificity, but this appears unlikely in this study for a number of reasons. The human and predicted macaque sequences share greater than 90% homology, with only 16 out of the 254 residues different between the two molecules. Human and mouse 4-1BBL sequences only have around 40% homology, yet we were able to detect an effect of human 4-1BBL in mice (Figure 5B), albeit reduced compared to mouse 4-1BBL. In addition, T cell responses against human 4-1BBL were observed in mice vaccinated with ChAd63.h4-1BBL, yet in macaques no T cell responses against human 4-1BBL peptides were seen, suggesting the two species share sufficient homology not to break self-tolerance.

A more likely explanation for the absence of an effect in non-human primates is the overall level of 4-1BBL that is required to induce a positive effect, be it the total level expressed or duration of the signal. In the two previous non-human primate studies agonist 4-1BB antibody was administered for 2 or 3 days, at various times during the effector phase of the response [27], [29]. While this approach could be replicated in our system by vaccination with ChAd63.h4-1BBL over several days, this vaccination regimen would only realistically be achieved for a therapeutic vaccine and not a vaccine which needs to align with the EPI vaccination regimen, in rural African settings.

An alternative approach to increase the adjuvant capacity of 4-1BBL from a vectored vaccine could be the co-administration with other costimulatory molecules from either the B7 or TNFR superfamily. The combination of multiple costimulatory signals may provide sufficient signalling to increase the response in higher order species, which appears difficult to reach by vaccination with a single molecular adjuvant. A combination of B7.1, LFA-3 and ICAM-1 (TRICOM) has been assessed both pre-clinically and clinically with a pox-viral platform against a number of different cancer models [47]-[49]. Indeed, two previous reports have shown enhancement of the response in mice by the use of murine 4-1BBL in combination with other costimulatory molecules when compared to the response when each was administered alone [50], [51]. More recently increased anti-viral efficacy of a HAdV5-gag vector in mice was observed by immunisation with a HAdV5 expressing a trimeric complex of murine 4-1BBL and murine BAFF fused to surfactant protein-D [52], further providing support for the use of 4-1BBL in a complex.

Despite the vast numbers of papers describing novel molecular adjuvants in murine models [53], many approaches have failed to translate into clinical vaccines. This is either due to the absence of an effect with other vector platforms or the absence or reduction of the effect in non-human primates, which often goes unreported. Having observed an enhancement in 3 vector systems, it appeared reasonable to anticipate that an effect with human 4-1BBL would have been achieved in rhesus macaques. So while the absence of an improvement in immunogenicity of our clinical vaccine, ChAd63.ME-TRAP, limits the translation of 4-1BBL in humans, this study highlights the importance of testing novel adjuvant approaches in higher order species.

## Materials and Methods

### Construction of DNA plasmids, recombinant adenoviruses and recombinant MVA vaccines

Bicistronic DNA plasmid vaccines utilizing tandem human cytomegalovirus immediate early promoters (phCMV) to express the model antigen TIP [37] and 4-1BBL were constructed as previously described [37].

To construct a recombinant MVA expressing three transgenes, namely TIP antigen, 4-1BBL, and a GFP marker gene, a mixture of PCR and conventional cloning was used to assemble the following elements in a plasmid suitable for insertion by recombination into the viral thymidine kinase locus: (i) GFP under control of the fowlpox virus p4B promoter; (ii) TIP under control of the vaccinia virus p7.5 promoter; and (iii) m4-1BBL under control of the short synthetic promoter. A control construct lacked the m4-1BBL ORF. The plasmid was transfected into MVA-infected primary chick embryo fibroblasts (CEFs; Institute for Animal Health, Compton, UK) and recombinant viruses were isolated by selection of GFP positive plaques, amplified, purified over sucrose cushions and titred in CEFs according to standard practise. The integrity, identity and purity of the viruses were confirmed by PCR analysis.

Construction of recombinant HAdV5 employed a Gateway-compatible entry vector similar to the DNA plasmid vaccine construct (above), but the TIP antigen was fused in-frame to the N-terminus of GFP, and the phCMV promoter driving its expression was replaced with the human elongation factor 1α promoter (phEF1) from pEF-BOS [54]. The m4-1BBL ORF was then conventionally cloned into the polylinker to place it under control of the remaining phCMV, positioned 5′ to the antigen expression cassette (a control construct lacked the m4-1BBL ORF). The resulting plasmids were inserted into the E1 locus of pAd/PL-DEST (Invitrogen) by *in vitro* recombination using LR clonase (Invitrogen). Expression cassettes were inserted into the E1 locus of a genomic clone of ChAd63 using homologous recombination in BJ5183 *E. coli* as previously described [55].

### Ethics Statement

Mice were used in accordance with the UK Animals (Scientific Procedures) Act under project license number 30/2414 granted by the UK Home Office. Animals were group housed in IVCs under SPF conditions, with constant temperature, humidity with lighting on a 12∶12 light-dark cycle (8am to 8pm). For induction of short-term anaesthesia, animals were either injected intraperitoneally (i.p.) with ketamine and Domitor or anaesthetised using vaporised IsoFlo. All animals were humanely sacrificed at the end of each experiment by an approved Schedule 1 method.

Ethical approval for use of rhesus macaques was granted by the University of Wisconsin-Madison IACUC (termed Animal Care and Use Committee) and was assigned protocol number g00615. The Wisconsin National Primate Research Center (WNPRC) executed the study to honor the fee-for service agreement between the University of Wisconsin, USA and the University of Oxford, UK. Processing of blood samples and fresh ELISpot were performed at the University of Wisconsin, with serum and frozen PBMCs shipped to Oxford for additional immunological investigations. Macaques were housed in standard stainless steel primate cages (Surburban Surgical, Chicago, IL): Animals were pair housed, or singly house when no compatible partners were available. All animals had visual and auditory contact with other monkeys in the same room. They were fed twice daily with commercial chow (20% protein primate diet, catalog no. 2050; Harlan Teklad, Madison, WI) and also given a variety of fruit enrichment in the afternoons. Housing rooms were maintained at 18°C to 24°C (65 to 75°F), 30 to 70% humidity, and on a 12∶12 light-dark cycle (on, 6∶00 a.m.; off, 6∶00 p.m.). As a source of environmental enrichment puzzle feeders of various sorts were provided at least twice per week, destructible foraging one time per week in addition to daily fruit or yoghurt cup.

All procedures were performed by highly trained animal technicians or veterinary staff and to minimize distress and suffering during vaccination or blood withdrawal, animals were anesthetized for all procedures. Blood draws were limited to the maximum volume allowed over the duration of the experiment. After each vaccination macaques were monitored for 14 days for behavioural changes, body temperature and redness or swelling at the sight of injection, no adverse response were observed, with only transient inflammatory responses (lasting between 24 and 48 hours) observed in 6 animals. All animals were returned into the colony at the end of the study for use in other experiments or breeding programs (as appropriate).

### Animals and Immunizations

Female Balb/c or C57BL/6J mice 6 weeks of age or older (Harlan, UK), were immunized intramuscularly (i.m.) in the musculus tibialis or intradermally (i.d.) in the ear with a total volume of 50 µl of vaccine diluted in PBS. For DNA immunizations, mice received 50 µg of DNA per immunization, MVA vaccinations mice received 10^6^ plaque forming units (PFU) per immunization and Adeno virus vaccines doses were based on infectious units (iu) and ranged from 10^8^ to 10^6^ iu per animal. Each experiment presented in this manuscript is representative of at least 2 experiments. Due to low levels of immunogenicity induced by DNA vaccination, immune response were measured in the spleen after two doses of DNA administered two weeks apart. To correspond to the peak in immunogenicity after viral vector vaccination [41], immune responses were measured in the spleen approximately 1 week after MVA and 2 weeks after HAdV5 vaccination.

Male rhesus macaques (*Macaca mulatta*) between 4 ½ to 8 ½ years were selected for use in this study based on negativity for SIV, STLV and SRV, had not previously received immunosuppressive treatment, vaccination with a malaria related antigen or a DNA, poxvirus or adenovirus vector and were in otherwise good health as assessed by the veterinarians of the WNPRC on the basis of physical examinations, blood chemistry and complete blood cell count results. In addition, subjects were pre-screened and excluded if they has high neutralising antibodies against ChAd63 and or background ELISpot responses to TRAP and ME peptides. Assignment of the final 12 subjects into each sub-group ensured an equal age and weight distribution between the two groups. The control group (ChAd63 ME-TRAP group) included a total of 6 animals with an average weight of 9.7 kg (range 7.9–13.5 kg) and age of 6 years (range 4.7–8.2); the test group (ChAd63.ME-TRAP and 5× ChAd63.h4-1BBL) included 6 animals with an average weight of 9.4 kg (range 7.3–13.3) and average age of 6 years (range 4.8–8.2). Animals received 2.75×10^8^ iu of ChAd63.ME-TRAP (1.01×10^10^ vp) alone or mixed with 1.38×10^9^ iu ChAd63.h4-1BBL (2.14×10^11^ vp) in a total of 0.3 ml into the left deltoid muscle. Blood samples were taken on the day of vaccination and at weeks 2, 4 and 8 weeks after vaccination.

### Antigens

The synthetic epitope string TIP [37], contains the H-2K^d^ restricted *P.berghei* circumsporozoite protein CD8^+^ T cell epitope Pb9 SYIPSAEKI (aa 252–260) [42], H-2d *Mycobacterium tuberculosis* antigen 85A CD4^+^ T cell epitope P15 TFLTSELPGWLQANRHVKPT (aa 142–161) [56], [57], H-2K^b^ CD8^+^ T cell epitope from SIV-gag AL11 AAVKNWMTQTL [58] (all purchased from Proimmune). To enable detection of antibodies from HAdV5 vectors, the TIP antigen was fused to EGFP, providing an additional H-2K^d^ restricted CD8^+^ T cell epitope HYLSTQSAL [59] (Proimmune). Responses to *P. falciparum* MSP-1 was measured against a pool containing H-2b defined CD4^+^ epitope M188 (DKIDLFKNPYDFEAIK) and a pool containing CD8^+^ T cell epitopes M86 (IPYKDLTSSNYVVKD), M100 (INDKQGENEKYLPFL) and M149 (YRSLKKQIEKNIFT) [60], *P. falciparum* AMA-1 with a pool of the dominant H-2d epitopes A31 (VFGKGIIIENSKTTF), A41 (FYKDNKYVKNLDELT), A42 (KYVKNLDELTLCSRH) and A95 (NKKIIAPRIFISDDK) [61], [62] and *P. falciparum* circumsporozoite protein (CS) with the dominant H-2K^d^ CD8^+^ T cell epitope CS_39–47_ (NYDNAGTNL) [63]. Responses to 4-1BBL were measured against a single pool containing 15 mer peptides (overlapping by 5) spanning the length of either mouse 4-1BBL (GenBank accession no. NP_033430) or human 4-1BBL (GenBank accession no. NP_003802) (Proimmune). A single pool containing all ME peptides together with two separate peptide pools (20 mers peptides overlapping by 10) spanning the length of TRAP from the T9/96 *P. falciparum* strain [7], were used to stimulate macaque PBMCs (Peptide Protein Research).

### ELISpots

For detection of murine antigen specific IFN-γ producing cells, ammonium chloride-potassium (ACK) lysis buffer treated mouse splenocytes or PBMCs were stimulated with the relevant peptide (final concentration 1–2 µg/ml) for 18–20 hours on IPVH-membrane plates (Millipore) coated with 5 µg/ml anti-mouse IFN-γ (AN18) (Mabtech). IFN-γ spot forming cells (SFC) were detected after staining membranes with anti-mouse IFN-γ biotin (1 mg/ml) (R46A2) (Mabtech) followed by streptavidin-Alkaline Phosphatase (1 mg/ml) (Mabtech) and development with AP conjugate substrate kit (BioRad, UK) and enumerated using an ELISPOT Reader System ELR04 (AID GMbH). Samples were plated in duplicate and the final data is presented as spot forming units (SFU) per million cells after subtracting the background (number of IFN-γ SFU in wells containing cells and media only).

Nonhuman primate specific IFN-γ ELISPOT assays were performed as previously described [64] using precoated ELISpot^PLUS^ kits according to the manufacturer’s recommendation (Mabtech USA, Mariemont, OH, USA). All tests were performed in duplicate using pools of 20-mers at a final concentration of 5 µg/ml and incubated for 12–18 hours at 37°C in 5% CO_2_. Wells were imaged and spots counted with an AID elispot reader ELR04 and AID elispot reader software V4.0. Background (mean of wells without peptide) levels were subtracted from each well on the plate. A response was considered positive if the mean number of SFCs of duplicate sample wells exceeded background plus two standard deviations (STD) and was >50 SFC per 1×10^6^ cells.

### ELISA

Antibodies induced by vaccination were measured by ELISA. Briefly, 96 well Nunc-Immuno Maxisorp plates were coated for 1 hour at room temperature with recombinant GFP (Millipore) at a concentration of 2 µg/ml diluted in bi-carbonate buffer (Sigma Aldrich). After blocking with 1% BSA in 0.5% Tween-20 PBS, serum was incubated for 2 hours prior to detection of bound antibodies with alkaline phosphatase-conjugated goat anti-mouse IgG (whole molecule) (Sigma Aldrich) diluted 1∶5000 and development with NPP substrate (Sigma Aldrich). Serum antibody endpoint titres were taken as the x-axis intercept of the dilution curve at an absorbance value 2× standard deviations greater than the OD_405_ for naïve mouse serum (typical cut-off OD_405_ for positive sera = 0.15). Serum from naïve mice were pooled and used as controls for all the ELISAs and were always below the level of detection.

### Intracellular cytokine staining

ACK lysis buffer treated splenocytes were stimulated at 37°C for a total of 6 hours with 1 µg/ml Pb9 peptide and 1 µg/ml Golgi-plug (BD) with the addition of CD107a-PE for the culture period when investigated (Figure 3b). Following surface staining for CD4-e450 and CD8-PerCPCy5.5 (all eBioscience), cells were fixed with 10% neutral buffered formalin solution (containing 4% formaldehyde) (Sigma) for 5 minutes prior to intracellular staining for TNF-α Alexa488, IL-2 PE and IFN-γ Alexa647 (all eBioscience) diluted in Perm-Wash buffer (BD).

Sample acquisition was performed on LSR II (BD) or Cyan (Beckman Coulter) and data analyzed in FlowJo (TreeStar). All graphs and statistical analysis were performed using Prism v5.0d (Graphpad) with final figures created in Adobe Illustrator CS5 v15.0.2 (Adobe).

### Malaria Sporozoite Challenge

*Plasmodium berghei* (ANKA strain clone 234) sporozoites (spz) were isolated from salivary glands of female *Anopheles stephensi* mosquitoes with each mouse receiving a total of 1,000 spz via the intravenous (i.v.) route. Mice were monitored daily from day 5 onwards by taking thin blood smears and staining with Giemsa (Sigma Aldrich) to detect the presence of schizonts within the red blood cells. Parasitaemia was calculated as the percentage of infected red blood cells per microscope field, with at least 5 fields counted per mouse per day. Using linear regression, the time to 0.5% parasitaemia was calculated based on the y-intercept and slope of the line. Mice were sacrificed by a Home Office approved Schedule 1 method after 4 consecutive positive thin blood films or parasitaemia above 1% (typically by day 8). As this low level of *P.berghei* parasitaemia mice do not typically display systemic signs of being unwell (eg ruffled fur, hunching). Sterile protection from sporozoite challenge was defined as the complete absence of parasite in the blood for more than 10 days.

## Supporting Information

Figure S1 **Genetic instability of adenovirus vectors containing dual CMV major immediate early promoters.** (**A, B**) Schematics of genetically unstable bi-cistronic gene expression cassettes inserted into the E1 locus of ChAd63. (**A**) Tandem arrangement of human cytomegalovirus major immediate-early promoters (hCMV), either wild-type intron A containing (iA) or with hybrid intron (Hi). (**B**) Tandem arrangement of hCMV with the simian cytomegalovirus immediate-early IE94 promoter (sCMV). Other features are the ME-TRAP and GFP open reading frames and the SV40 and BGH polyadenylation signals (pA). (**C, D, E**). Restriction endonuclease analysis showing viral genetic instability. Viral genomic DNA (V) was isolated from CsCl-banded virus after three serial viral passages in HEK293 cells and compared to pre-viral plasmid (P). (**C**) Dual hCMV digested with *Pme*I and *ApaL*I. (**D**) hCMV-sCMV digested with *Pme*I and *ApaL*I. (**E**) hCMV-sCMV digested with *Pme*I and *Not*I. Sizes (bp) of marker bands are indicated on the left. Other sizes indicate the restriction fragments containing the transgenic expression cassette. *Pme*I liberates the left end of the genome from the pre-viral plasmid; *ApaL*I cuts 3′ to the transgene cassette in the viral genome; and there are *Not*I sites immediately 5′ of each poly A signal. Asterisks indicate aberrant bands arising from recombination between the identical or homologous CMV promoters. In panel E it can be seen that this recombination has resulted in the almost complete loss of the 1,963 bp band containing the ME-TRAP open reading frame.
(TIF)

Figure S2 **Adjuvant effect of 4-1BBL in a prime-boost vaccine regimen. A**) Balb/c mice were vaccinated i.m. with 50 µg of DNA plasmid pIMM.TIP or pIMM.TIP 4-1BBL and boosted two weeks later with 10^6^ PFU of MVA.TIP or MVA.TIP 4-1BBL. Spleens were harvested a further 2 weeks after vaccination and the response to Pb9 measured by IFN-γ ELISpot. Data was analysed with a Kruskal-Wallis test of variance but no significant effect of vaccination regimen was observed. **B**) Balb/c mice were vaccinated i.m. with 10^6^ iu HAdV5.TIPEGFP or HAdV5.TIPEGFP 4-1BBL and boosted 8 weeks later i.d. with 10^6^ PFU MVA.TIP or MVA.TIP 4-1BBL. Spleens were harvested a further two weeks later and response to Pb9 measured by IFN-γ ELISpot. Bars represent the median with each animal displayed as a single point. Data was analysed with a Kruskal-Wallis test of variance but no significant effect of vaccination regimen was observed.
(TIF)

Figure S3 **ELISpot response to mouse and human 4-1BBL peptides.** Balb/c mice were vaccinated i.m. with 10^6^ iu ChAd63.ME-TRAP and either 5 or 10× ChAd63.m4-1BBL (black squares) or ChAd63.h4-1BBL (grey squares). Spleens were harvested two weeks later and response to either mouse (**A**) or human (**B**) 4-1BBL peptides measured by IFN-γ ELISpot. Bars represent the median with each animal displayed as a single point.
(TIF)
