# Supplementary figures and images for: 4-1BBL Enhances CD8+ T Cell Responses Induced by Vectored Vaccines in Mice but Fails to Improve Immunogenicity in Rhesus Macaques
Source: PLoS One. 2014 Aug 20;9(8):e105520. doi: 10.1371/journal.pone.0105520 (PMC4139357; doi:10.1371/journal.pone.0105520)

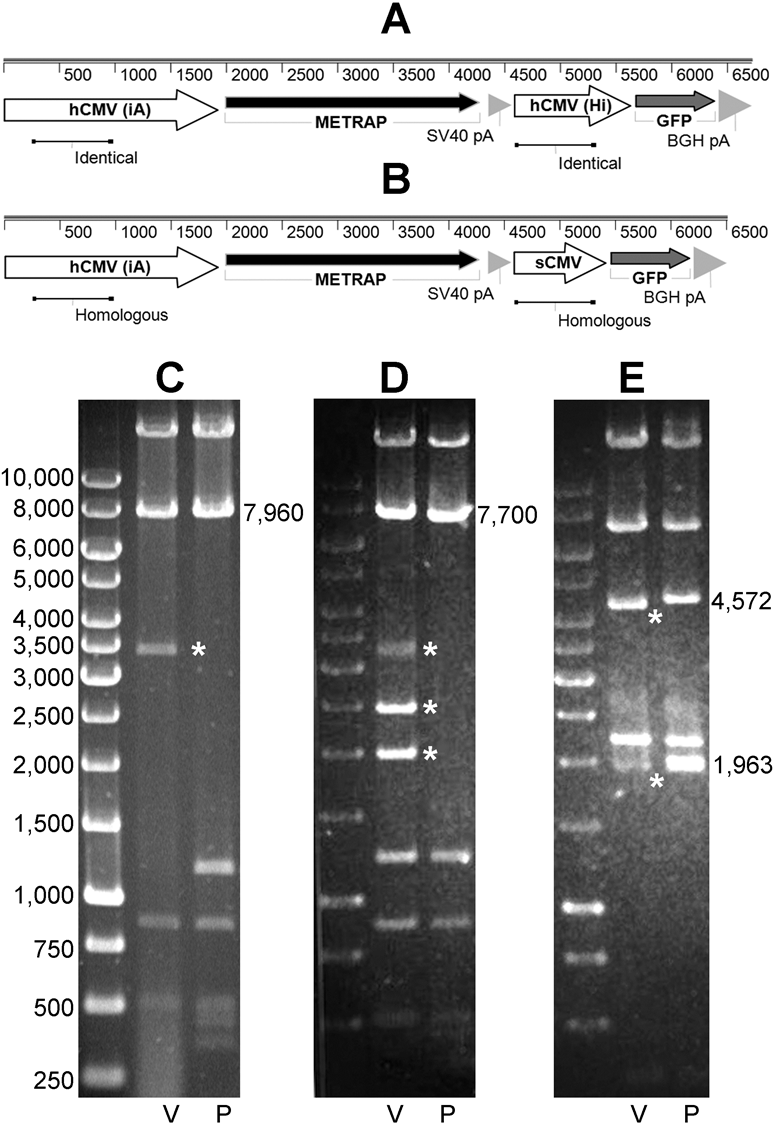

Supplement: Figure S1 — Genetic instability of adenovirus vectors containing dual CMV major immediate early promoters. (A, B) Schematics of genetically unstable bi-cistronic gene expression cassettes inserted into the E1 locus of ChAd63. (A) Tandem arrangement of human cytomegalovirus major immediate-early promoters (hCMV), either wild-type intron A containing (iA) or with hybrid intron (Hi). (B) Tandem arrangement of hCMV with the simian cytomegalovirus immediate-early IE94 promoter (sCMV). Other features are the ME-TRAP and GFP open reading frames and the SV40 and BGH polyadenylation signals (pA). (C, D, E). Restriction endonuclease analysis showing viral genetic instability. Viral genomic DNA (V) was isolated from CsCl-banded virus after three serial viral passages in HEK293 cells and compared to pre-viral plasmid (P). (C) Dual hCMV digested with PmeI and ApaLI. (D) hCMV-sCMV digested with PmeI and ApaLI. (E) hCMV-sCMV digested with PmeI and NotI. Sizes (bp) of marker bands are indicated on the left. Other sizes indicate the restriction fragments containing the transgenic expression cassette. PmeI liberates the left end of the genome from the pre-viral plasmid; ApaLI cuts 3′ to the transgene cassette in the viral genome; and there are NotI sites immediately 5′ of each poly A signal. Asterisks indicate aberrant bands arising from recombination between the identical or homologous CMV promoters. In panel E it can be seen that this recombination has resulted in the almost complete loss of the 1,963 bp band containing the ME-TRAP open reading frame. (TIF) [file pone.0105520.s001.tif]

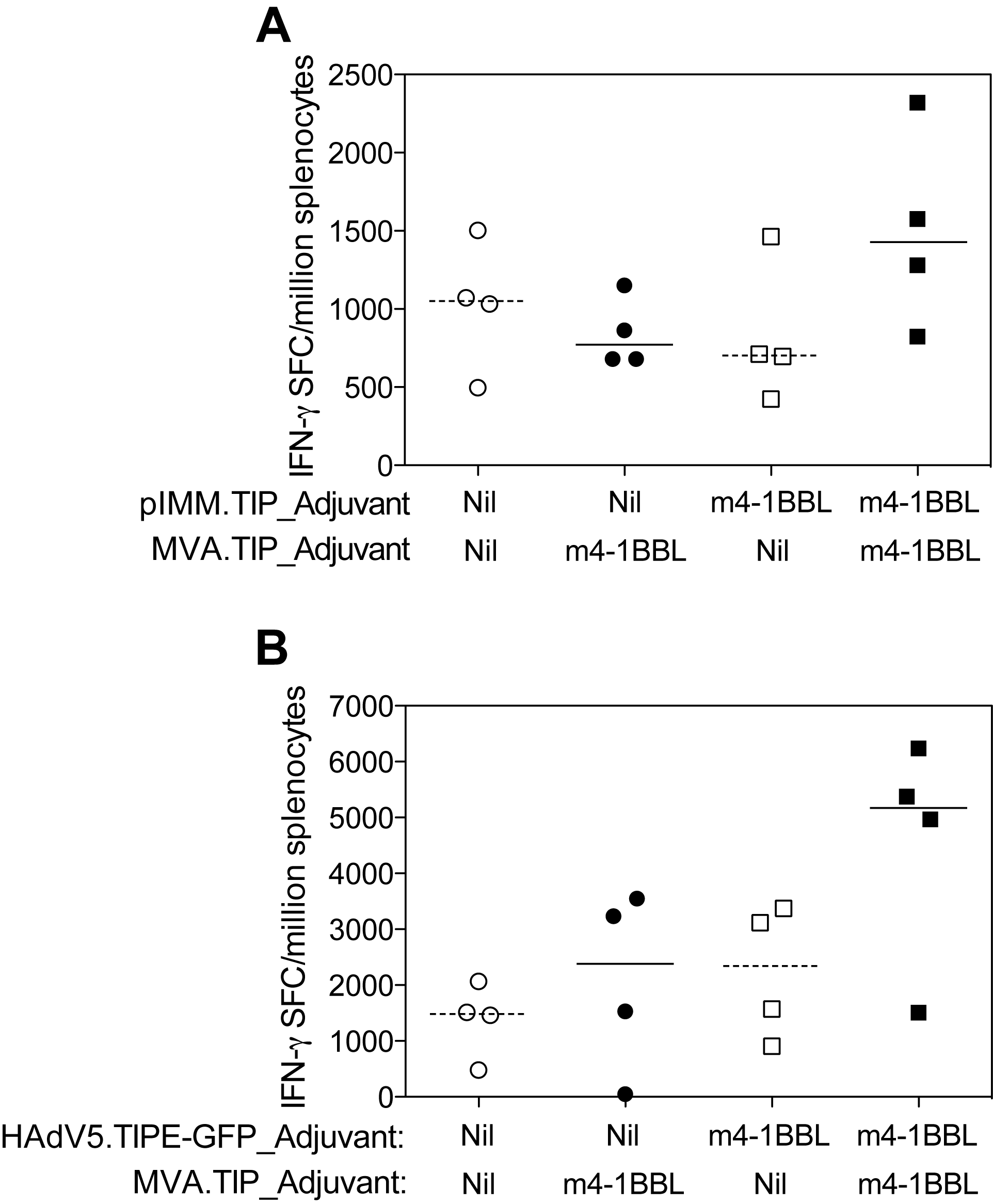

Supplement: Figure S2 — Adjuvant effect of 4-1BBL in a prime-boost vaccine regimen. A) Balb/c mice were vaccinated i.m. with 50 µg of DNA plasmid pIMM.TIP or pIMM.TIP 4-1BBL and boosted two weeks later with 106 PFU of MVA.TIP or MVA.TIP 4-1BBL. Spleens were harvested a further 2 weeks after vaccination and the response to Pb9 measured by IFN-γ ELISpot. Data was analysed with a Kruskal-Wallis test of variance but no significant effect of vaccination regimen was observed. B) Balb/c mice were vaccinated i.m. with 106 iu HAdV5.TIPEGFP or HAdV5.TIPEGFP 4-1BBL and boosted 8 weeks later i.d. with 106 PFU MVA.TIP or MVA.TIP 4-1BBL. Spleens were harvested a further two weeks later and response to Pb9 measured by IFN-γ ELISpot. Bars represent the median with each animal displayed as a single point. Data was analysed with a Kruskal-Wallis test of variance but no significant effect of vaccination regimen was observed. (TIF) [file pone.0105520.s002.tif]

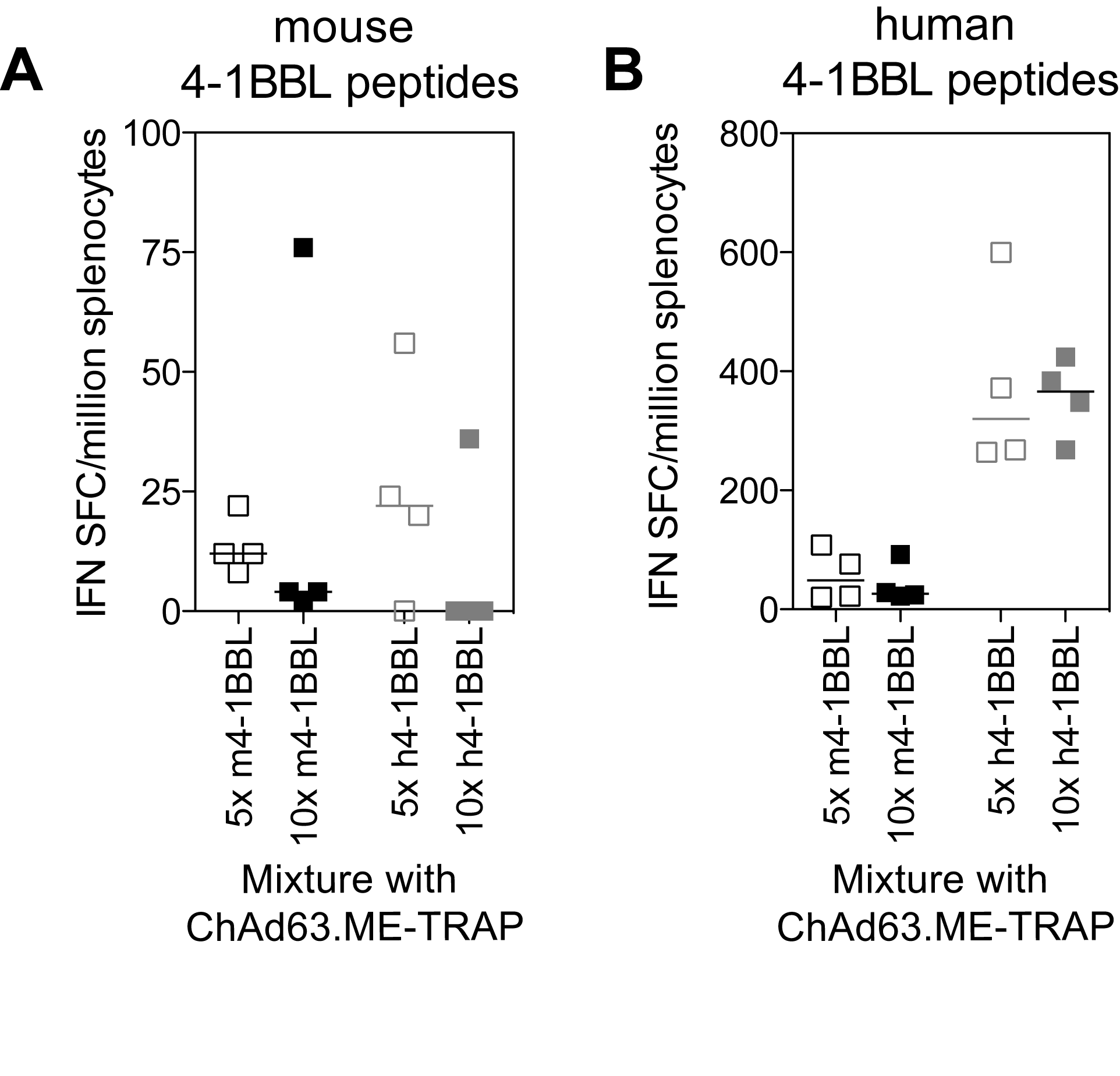

Supplement: Figure S3 — ELISpot response to mouse and human 4-1BBL peptides. Balb/c mice were vaccinated i.m. with 106 iu ChAd63.ME-TRAP and either 5 or 10× ChAd63.m4-1BBL (black squares) or ChAd63.h4-1BBL (grey squares). Spleens were harvested two weeks later and response to either mouse (A) or human (B) 4-1BBL peptides measured by IFN-γ ELISpot. Bars represent the median with each animal displayed as a single point. (TIF) [file pone.0105520.s003.tif]
